# Microwave Modification as an Excellent Way to Produce Unique Lysozyme with Potential for Food and Human Health

**DOI:** 10.3390/foods10061319

**Published:** 2021-06-08

**Authors:** Tianyu Yang, Grzegorz Leśnierowski

**Affiliations:** Department of Food Safety and Quality Management, Faculty of Food Science and Nutrition, Poznan University of Life Sciences, Wojska Polskiego 31, 60-624 Poznan, Poland; grzegorz.lesnierowski@up.poznan.pl

**Keywords:** lysozyme, microwave modification, oligomerization, hydrophobicity and utility, β-interferon

## Abstract

Thermal modification is an effective method of converting lysozyme into a protein with a very strong bactericidal effect and a broad multidirectional application potential. In this research, we used our innovative method, which involves the use of microwave radiation, in the lysozyme modification process. With the optimization of modification conditions as the main purpose of the study, we focused on the assessment of the impact of a strongly acidic modification environment and the duration of modification on the effectiveness of the modification of lysozyme. Moreover, an innovation was introduced and included the use of a commercially produced liquid lysozyme concentrate (LLC) as the starting material instead of the dried form that is commonly used. The obtained results showed that the modified preparations contained an abundant amount of oligomers, and the surface hydrophobicity increased by more than 50% compared to the native form of the enzyme, which means that the modification had a strong influence on the effectiveness of the antibacterial activity. Moreover, these changes made it possible to obtain preparations with complete solubility, which could not be obtained with other thermal methods. Thus, using microwave radiation to modify LLC is a unique and effective modification scheme because it can obtain the highest quality of a large number of attractive products in a simple and fast way with multidirectional application potential, especially in food technology, medicine, pharmacology, and veterinary medicine.

## 1. Introduction

Hen egg white lysozyme (E.C.3.2.1.17), as an enzyme, has a strong bactericidal effect on Gram-positive bacteria, destroying them by the hydrolysis of the β-1,4 glycosidic bonds located in their cell wall. However, the hydrolysis of this bond by lysozyme in Gram-negative bacteria is difficult and, in most cases, even impossible due to the presence of additional layers in the wall of these bacteria that protect the bond and thus prevent the enzyme from accessing it. Hence, the effect of native lysozyme on these bacteria is limited [1]. However, this obstacle can be overcome by using modified enzyme, which, in its new form, is able to reach and destroy this sensitive binding that the native enzyme cannot. To obtain this form of lysozyme, it must therefore be modified, which can be achieved in various ways. The most commonly used and best-known methods are thermal and thermochemical methods. Previous studies have shown that regardless of the modification method used, it always results in the oligomerization of lysozyme and changes in the hydrophobicity distribution between its internal and external parts [2,3,4,5]. These properties mean that the modified enzyme can be widely used in many industries, particularly the food industry, to protect and extend the shelf life of various types of food.

Among the various methods of lysozyme modification, thermal methods are considered the most important, and this type of method also includes the innovative method of lysozyme modification based on the use of microwave radiation. Preliminary studies on this method have already demonstrated many of its advantages [6,7], such as the ease of carrying out the modification process, the short duration, and the high degree of enzyme modification. The possibility of obtaining an attractive product with high application potential in a relatively easy and inexpensive way prompted us to continue working on this method, and the first goal of the research presented in this paper was to further optimize the conditions of the modification process. The influence of the strong acid modification medium application and the process duration on the effectiveness and quality of the obtained product were investigated. The second goal was to evaluate the feasibility of using the more commonly commercially produced liquid lysozyme concentrate as the raw material instead of the previously used dried lysozyme in the form of a powder, which could be modified only after its appropriate preparation.

## 2. Materials and Methods

### 2.1. Materials

The material for modification was a liquid lysozyme concentrate that was commercially produced and distributed by SACCO Polska (Kościan, Poland). The lysozyme monomer content in the concentrate was 20% and its activity was 8804 U/µL. Liquid lysozyme concentrate (LLC) was characterized by high chemical purity; the total protein content was over 94%.

### 2.2. Modification of LLC 

Prior to modification, the LLC stock solution was diluted 2-fold so that the lysozyme concentration in the modified solution was 10%. This solution was prepared for modification by adjusting its pH to 2.0, 3.0, and 4.0 with hydrochloric acid or sodium hydroxide (with the appropriate concentration: 1.0 or 0.5 M) and 1.5% benzodiazepine as a protective substance against the effects of high temperature. Ten milliliters of the prepared solutions (pH 2, 3, and 4) were placed in appropriate pressure containers that were carefully sealed and modified in the microwave integrator (Sharp device model R-978-A) at 270 W (frequency of 2.45 GHz) for 180, 200, 220, 240, and 260 s. After the modification was completed, the samples were cooled to 0 to 1 °C, and then hydrogen peroxide was added as an oxidizing agent (in such an amount that its concentration in the solution was 1.5%). The modified samples were stored at 4–6 °C.

### 2.3. Electrophoretic Analysis

The electrophoretic analysis was carried out according to the method of Laemmli (1970) with modifications by Leśnierowski (2007) concerning the analysis of the composition of various forms of lysozyme [2,8]. In this research, electrophoretic separation was carried out using Hoefer Scientific SE-600 apparatus (SE-600, Hoefer, Inc., Holliston, MA, USA) at a current of 60 mA in a 6% polyacrylamide thickening gel and then at a current of 90 mA in a 12.5% separation gel.

After fractionation was completed, the gels were fixed and stained according to the procedure and then scanned into computer files and densitometric analysis was performed using TotalLab Quant professional software from Nonlinear Dynamics Ltd. (Newcastle up on Tyne, UK, Milford, MA, USA).

### 2.4. Hydrolytic Activity 

The hydrolytic activity of the samples was determined by the spectrophotometric method according to the procedure given by Leśnierowski (2007) [2], the principle of which is to measure the degree of turbidity reduction in the bacterial suspension (Micrococcus lysedeikticus) after adding lysozyme to the suspension solution using the VSU2-P spectrophotometer by Carl Zeiss Jena (VSU2-P, Oberkochen, Germany).

The final results are expressed as the residual hydrolytic activity (RHA), which is the percentage of the measured activity of the modified LLC HAL1 to the activity of the natural LLC monomer HAL0, according to the following formula:

RHA = (HALi/HAL0) × 100 (%)

where RHA is the residual hydrolytic activity; HALi is the hydrolytic activity of modified LLC, for subsequent samples “i”; and HAL0 is the hydrolytic activity of natural LLC.

### 2.5. Hydrophobicity 

The hydrophobicity measurement method was based on the procedure of Lieske and Konrad (1994) [9].

According to this procedure, the test samples were diluted to a concentration of 0.1% with a phosphate buffer solution at pH 6.5. Then, to the four test tubes marked a, b, a’, and b’, the following were added successively:

(1) For test tube a: 50 µL of lysozyme and 50 µL of distilled water;
(2) For test tube a’: 50 µL of lysozyme and 50 µL of 0.25% Tween 80 solution;
(3) For test tube b: 100 µL of distilled water;
(4) For test tube b’: 50 µL of distilled water and 50 µL of 0.25% Tween 80 solution.

To each of these tubes, 2.5 mL of the Bio-Rad dye with a concentration of 0.02% was added, thoroughly mixed, and left for 16 min until the color fully developed. Then, the absorbance of the solutions was measured at λ = 595 nm (VSU2-P, Carl Zeiss Jena, Germany) and the surface hydrophobicity SH was calculated from the following formula:

$$SH=\frac{\left[a-b\right]-\left[a^{\prime }-b\prime \right]}{\left[a-b\right]}\times 100\%$$

The changes in the surface hydrophobicity as the ∆SH in modified LLC preparations are provided. According to the formula presented by Leśnierowski (2007) [2], ∆SH is the difference in the surface hydrophobicity of the SHLi samples and the native (unmodified) LLC SHL0 lysozyme sample:

∆SH = SHLi − SHL0

where ∆SH is the change in surface hydrophobicity; SHL0 is the native LLC surface hydrophobicity; and SHLi is the surface hydrophobicity of modified LLC, for subsequent samples “i”.

### 2.6. Statistics Analysis

The results were subjected to statistical analysis using STATISTICA version 13.1 software (TIBCO Software Inc., Hillview, CA, USA). The results are expressed as the means ± standard deviations. An analysis of variance was performed. The Fisher’s least significant difference (LSD) test was used to detect significant differences of results at α = 0.05. Linear regression analysis was also performed.

## 3. Results

### 3.1. Presenting Characteristics

**Figure 1:**
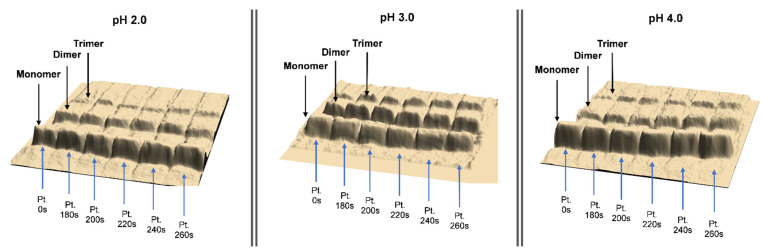
Modification of LLC by the microwave method. Electrophoretic images of the enzyme after modification at pH 2.0, 3.0, and 4.0 and process duration times of (Pt) 0, 180, 200, 220, 240, and 260 s.

The preparations obtained after the modification of the enzyme carried out in new conditions in accordance with the procedure described in the section “Materials and Methods” were subjected to analytical tests. All obtained data are presented in Table 1, while Figure 1 shows the electropherograms illustrating the effect of enzyme oligomerization.

### 3.2. Analyzing Enzyme Oligomerization and Hydrolytic Activity

The presented data clearly show that both parameters of this process, i.e., the time of the process and pH, had a significant impact on both the oligomerization and changes in hydrolytic activity in the preparations obtained after modification, as well as changes in the enzyme surface hydrophobicity. The data show that the lower the sample acidities and the longer the modification time used, the larger the number of dimers and trimers formed. To determine the observed relationships between these factors, a linear regression analysis of oligomer formation was performed, the results of which are presented in Table 2. 

The *p*-value for each factor suggests that the factors influencing total oligomer formation have a statistically significant effect. Based on the obtained data of the linear regression analysis, it can be assumed that for the applied enzyme modification conditions, the total number of oligomers will be formed in accordance with the obtained equation:

Sum of all oligomers = 21.18 − 2.66 × pH + 0.08 × modification time

The absolute value of the comparison of the normalized regression coefficients shows that the modification time factor (absolute value: 0.88) under these conditions exerts a more effective influence on the effects of modification than that of the pH factor (absolute value: 0.28). Moreover, the regression coefficient for the pH parameter below 0 for such a well-matched formula also confirms that an increase in pH above 4 has an inhibitory effect on the production of oligomers in the obtained preparations.

### 3.3. Analyzing Changes in Surface Hydrophobicity

An analogous analysis performed for the changes in the hydrophobic surface of the modified enzyme (the results of which are shown in Table 3) showed similar relationships. It was noted that the action of the oxidizing agent itself (H_2_O_2_ addition) without the use of microwave radiation caused a slight increase in surface hydrophobicity in the samples: for a pH of 4.0, this area increased by 10.75%, and for a pH of 2.0, the hydrophobicity increased by 13.82%. However, a significant increase in surface hydrophobicity took place only after the microwave process when it increased by up to 50%. The tendency of the observed changes depended not only on the acidity of the samples but also on the duration of the modification. The ∆SH values were higher when the pH value was lower, and the modification time was longer. To determine the significance of the influence of both factors, as in the case of oligomerization, a two-way regression analysis was performed, and the obtained data (Table 3) showed that, in this case, the modification time (absolute value of standardized regression coefficient: 0.93) was also more important than the acidity (absolute value of standardized regression coefficient: 0.29) of the samples. The conducted analysis also allowed us to determine the formula enabling the estimation of changes in hydrophobicity depending on the changing conditions of enzyme modification, which took the form of

∆SH = 22.58 − 3.78 × pH + 0.11 × Modification time

## 4. Discussion

### 4.1. Characteristics of the New Method

The research results presented in this paper are a continuation of research on a new method of the modification of lysozyme carried out in an oxidizing environment with the use of microwave radiation as the main modifying factor. The main purposes of these studies were, first, to optimize selected factors of the lysozyme modification process with the assessment of its main physicochemical properties and, second, to verify the possibility of using LLC as a raw material for enzyme modification. Previous studies showed that the use of a hybrid modification method (combination of microwave energy with oxidation) led to the formation of a new oligomeric form of lysozyme with a significant increase in its hydrophobic surface [6,7]. It has been shown that for a wide range of pH values of the environment used, from acidic (pH 4.0) to alkaline (pH 8.0), the acidic pH was more favorable because a better modification effect was then observed [6]. In other studies on the impact of the applied microwave power [7], it was proven that increasing the value from 270 to 630 W did not have a significant influence on the effectiveness of the modification. However, importantly, it was observed that the higher the power applied, the higher the tendency to denature the enzyme, which ultimately deteriorated the quality of the preparations obtained. Taking these data into account, in this study, the process was carried out with a constant power value of 270 watts, while in the strongly acidic environment used, the pH changed in the range from 2.0 to 4.0, and the modification time was from 180 to 260 s. As in the case in previous studies, the addition of benzodiazepine as a protective substance was also used to protect lysozyme against the destructive effects of drastic modification conditions. A significant procedural change in this work compared to all previous ones was the use of liquid lysozyme concentrate as the starting material for modification. In relation to the dried lysozyme powder used to date, it seems that the use of this material may have many advantages, such as a significant increase in the efficiency of the process, resulting in a 2-fold or even a 4-fold higher concentration of the enzyme in the raw material than that when using powder, as well as facilitation of the preparation of raw material samples for the modification process. In addition, the use of LLC is justified because lysozyme producers more often produce lysozyme in the form of concentrated solutions (with a lysozyme concentration of approx. 20%) than the dried product.

### 4.2. Consideration of the Physicochemical Properties of the Modified Lysozyme

The performed research showed that the use of LLC as the raw material in combination with the new specific conditions of the modification process gave interesting results. The preparations obtained after the modification, as expected, contained dimeric and trimeric forms in addition to the monomer. The obtained data indicate that the direction of changes in physicochemical properties was analogous to that observed in other thermal modifications when high-quality lysozyme preparations with an increased spectrum of antimicrobial activity were obtained and characterized by new and valuable functional properties [10,11]. In several aspects, the method described in this paper was superior to other known thermal and thermochemical modification methods of lysozyme. As previously shown in the preliminary work [6,7], the use of microwave radiation significantly shortened the time of enzyme modification, with the entire process lasting from 3 to a maximum of 4.5 min. Another advantage was the lack of even a trace of any insoluble precipitation resulting from, for example, enzyme denaturation processes, which are characteristic phenomena occurring during thermal modifications, especially those where temperatures above 60 °C are used. After microwave modification, the enzyme obtained was completely soluble (especially in the case of LLC). The prepared solutions did not contain any sediment, which greatly facilitated the practical use of the obtained preparations. Moreover, the use of LLC as a raw material also improved the modification efficiency. This effect was due to a two-times higher concentration of the enzyme in the modified solution (which may be up to four times higher), and, importantly, the obtained preparations still retained high-quality products. After analyzing the obtained data, we found that depending on the duration of the modification and the applied pH of the modified raw material, the total amount of oligomers in the preparations obtained after modification varied from 13.1 to 40.9%. Other methods of modifying lysozyme, especially high-thermal methods [12], allowed the production of preparations with more oligomers than was obtained using the microwave method. However, at the same time, these preparations contained partially denatured enzymes, so their practical utility was reduced. Therefore, in the case of the microwave method presented here, preparations were obtained, the usefulness of which may be even higher, despite the smaller amount of oligomers, because they do not contain even traces of the enzyme that is insoluble in aqueous solutions.

By analyzing another valuable quality feature of the enzyme, its hydrolytic activity has also been shown to change significantly under the modification conditions used. However, the observed relationships were opposite to those in the case of oligomerization, and it was found that, along with lowering the pH of the samples and extending the duration of the modification process, this activity was constantly reduced. It was noted that in the samples where the pH value was the lowest and the modification time was the longest, the amount of the remaining hydrolytic activity in relation to its value in the native enzyme was only 32%. However, even such a significant reduction was not surprising, as it is known from previous studies that any modification of lysozyme, regardless of the method used, always reduces this value. At the same time, in each case, lysozyme released its latent potential in the form of a new specific activity, which significantly increased the total antimicrobial activity (TAA) of the enzyme [11,13,14]. A characteristic feature of TAA is that the more oligomers (especially dimers) present in the modified lysozyme preparation, the greater the spectrum and the effectiveness of its antibacterial action. Although the mechanism of the new activity has not been determined or explained to date, it is believed that its occurrence depends on several factors, of which the degree of enzyme oligomerization is one of the most important [15,16]. On the other hand, it is claimed that the influence of changes in the location of the hydrophobicity in the enzyme molecule is an equally important factor in the formation of TAA [16,17]. This is a consequence of the structure of lysozyme because its molecule is hydrophilic on its surface, and the hydrophobic part is closed inside. As a result of the modification of the enzyme, along with the unfolding of the molecule, this hidden hydrophobicity is released and remains on its surface. It has been proven that in the case of this parameter, similar to oligomerization, an increase in its value, according to the hypothesis of Ibrahim et al. [3] and subsequent studies on the modified lysozyme [11,18], causes an increase its total antimicrobial activity. For this reason, this feature is a very important characteristic of the modified enzyme and is the basis for its qualitative evaluation.

Therefore, the analysis of the obtained results showed that the modification of LLC with the microwave method along with the reduction in the acidity of the modifying environment and the extension of the processing time led to the formation of preparations with an increasing degree of enzyme oligomerization and surface hydrophobicity. This is not surprising because, based on data from previous studies [6,7], the range of the applied pH was carefully selected and narrowed to the pH value from 2.0 to 4.0, and the time was extended to 260 s. Under the most favorable modification conditions, i.e., with a modification time of 260 s and a pH of 2.0, a preparation containing over 41% oligomers (28.3% dimer and 12.7% trimer) was obtained, and its surface hydrophobicity increased to 50%. Such values of its most important parameters indicate a very favorable and expected modification, despite a significant reduction in hydrolytic activity compared to the native form of the enzyme (reduction of approximately 70%). However, as previously emphasized, such transformation of lysozyme is accompanied by the emergence of a new specific form of activity, which, together with the remaining part of the catalytic activity, creates a kind of new potential in the form of TAA, which significantly extends the spectrum of the enzyme’s antimicrobial activity [18,19]. Therefore, the occurrence of this effect is very likely in this case, and confirmation of this phenomenon and extensive microbiological tests will be our main research direction in the next stage of research on the microwave method of lysozyme modification.

Another extremely important aspect related to the modified lysozyme, regardless of the type of modification used to obtain it, as presented in this work, is that it exhibits many other valuable properties, particularly those significant in medicine, pharmacology, and veterinary medicine [18]. Among the most important of them are the ability to synthesize tumor necrosis factor (TNFα) and the ability to stimulate the production and release of type I interferon (INFα, INFβ, and INFγ), interleukin-2 (II-2), and interleukin-6 (IL-6) by human lymphocytes [20]. Such action of the modified lysozyme, especially stimulating the formation of interferon in the human body, especially its beta type (INFβ), may significantly influence the strengthening of the immune system against various infections, including, according to the latest research, also against the SARS-CoV-2 virus [21,22].

### 4.3. Final Assessment

The newly presented results of research on the microwave method of lysozyme modification once again indicate that the hypothesis concerning the use of microwave radiation for enzyme modification is not only an interesting idea but also an exceptionally attractive and effective technology for the production of its modified form. As a result of the optimization of the modification conditions and the use of liquid lysozyme concentrate as a raw material for its operation, preparations with a high degree of oligomerization and a significantly enlarged hydrophobic surface were obtained. Under the most favorable conditions, these preparations contained dimers and trimers of over 40%, and the surface hydrophobicity increased by almost 50%, which indicates their high quality. A very important innovation of this method was the use of an LLC as a raw material for modification. Its use significantly increased the efficiency of the process, and the final product was completely soluble, without any traces of denatured particles, the presence of which was a negative feature of preparations obtained from the dried raw material. Thus, the presented method represents not only a way to obtain an attractive product with considerably useful potential but also a very attractive, easy-to-implement and effective tool for the industrial production of modified lysozyme, which should also be of interest to various egg-processing plants, especially those producing lysozyme in the form of a liquid concentrate.

## Figures and Tables

**Table 1:** Characterization of the physicochemical parameters of an LLC modified with the microwave method.

No.	pH	Duration Time (s)	Dimer (%)	Trimer (%)	Total Oligomers (%)	Residual Hydrolytic Activity (%)	Change in Surface Hydrophobicity *∆*SH (%)
1	2	0	13.47 ± 0.21 ^p^	5.20 ± 0.08 ^m^	18.67 ± 0.29 ^n^	68.61 ± 0.87 ^g^	13.82 ± 0.18 ^m^
2	2	180	20.00 ± 0.31 ^k^	7.35 ± 0.12 ^j^	27.35 ± 0.43 ^j^	54.60 ± 0.7 ^k^	32.25 ± 0.41 ^i^
3	2	200	21.12 ± 0.33 ^i^	7.96 ± 0.13 ^h^	29.08 ± 0.46 ^h^	45.25 ± 0.58 ^n^	35.33 ± 0.45 ^f^
4	2	220	24.69 ± 0.39 ^e^	10.00 ± 0.16 ^d^	34.69 ± 0.55 ^e^	38.76 ± 0.49 ^o^	39.42 ± 0.50 ^d^
5	2	240	26.22 ± 0.41 ^c^	11.53 ± 0.18 ^b^	37.75 ± 0.59 ^c^	33.88 ± 0.43 ^p^	45.65 ± 0.58 ^b^
6	2	260	28.26 ± 0.44 ^a^	12.65 ± 0.2 ^a^	40.91 ± 0.64 ^a^	31.88 ± 0.41 ^q^	49.88 ± 0.64 ^a^
7	3	0	10.41 ± 0.16 ^q^	4.18 ± 0.07 ^n^	14.59 ± 0.23 ^o^	83.38 ± 1.06 ^c^	12.12 ± 0.15 ^n^
8	3	180	16.53 ± 0.26 ^m^	7.14 ± 0.11 ^k^	23.67 ± 0.37 ^l^	75.62 ± 0.96 ^e^	29.86 ± 0.38 ^k^
9	3	200	17.45 ± 0.27 ^l^	7.55 ± 0.12 ^i^	25.00 ± 0.39 ^k^	66.48 ± 0.85 ^h^	32.83 ± 0.42 ^h^
10	3	220	21.53 ± 0.34 ^h^	9.08 ± 0.14 ^f^	30.61 ± 0.48 ^g^	57.30 ± 0.73 ^j^	35.33 ± 0.45 ^f^
11	3	240	24.08 ± 0.38 ^f^	10.30 ± 0.16 ^d^	34.38 ± 0.54 ^e^	51.82 ± 0.66 ^i^	38.63 ± 0.49 ^e^
12	3	260	27.04 ± 0.43 ^b^	11.63 ± 0.18 ^b^	38.67 ± 0.61 ^b^	49.58 ± 0.63 ^m^	41.26 ± 0.53 ^c^
13	4	0	10.00 ± 0.16 ^r^	3.06 ± 0.05 ^o^	13.06 ± 0.21 ^p^	91.66 ± 1.17 ^a^	10.75 ± 0.14 ^o^
14	4	180	15.10 ± 0.24 ^o^	6.63 ± 0.1 ^l^	21.73 ± 0.34 ^m^	88.31 ± 1.13 ^b^	27.33 ± 0.35 ^i^
15	4	200	16.53 ± 0.26 ^n^	7.14 ± 0.11 ^k^	23.67 ± 0.37 ^l^	80.08 ± 1.02 ^d^	29.35 ± 0.37 ^k^
16	4	220	20.30 ± 0.32 ^j^	8.06 ± 0.13 ^gh^	28.36 ± 0.45 ^i^	72.06 ± 0.92 ^f^	31.22 ± 0.40 ^j^
17	4	240	23.16 ± 0.36 ^g^	9.39 ± 0.15 ^e^	32.55 ± 0.51 ^f^	67.41 ± 0.86 ^h^	33.66 ± 0.43 ^g^
18	4	260	25.61 ± 0.4 ^d^	10.92 ± 0.17 ^c^	36.53 ± 0.57 ^d^	62.26 ± 0.79 ^i^	38.64 ± 0.49 ^e^

^a–r^ Different letters in columns denote a significant difference for means at *p* ≤ 0.05. Number of test repetitions: *n* = 5.

**Table 2:** Regression analysis results for sum of all oligomer formation during microwave modification of LLC.

Effect ^1^	b* ^2^	Standard Error of b*	b ^3^	Standard Error of b	t	*p*-Value
Intercept			21.18	1.40	15.12	0.000
pH	−0.28	0.04	−2.66	0.39	−6.76	0.000
Modification time (s)	0.88	0.04	0.08	0.004	21.37	0.000

^1^ R = 0.92; R^2^ = 0.85; adjusted R^2^ = 0.85. ^2^ b* is the standardized regression coefficient. ^3^ b is the non-standardized regression coefficient.

**Table 3:** Regression analysis results for total hydrophobicity formation during microwave modification of LLC.

Effect ^1^	b* ^2^	Standard Error of b*	b ^3^	Standard Error of b	t	*p*-Value
Intercept			22.58	1.01	22.35	0.000
pH	−0.29	0.02	−3.78	0.28	−13.34	0.000
Modification time (s)	0.93	0.02	0.11	0.002	42.20	0.000

^1^ R = 0.98; R^2^ = 0.96; adjusted R^2^ = 0.96. ^2^ b* is the standardized regression coefficient. ^3^ b is the non-standardized regression coefficient.

## Data Availability

The data presented in this study are available on request from the corresponding author.
